# Quinolone resistance in *Escherichia coli *from Accra, Ghana

**DOI:** 10.1186/1471-2180-11-44

**Published:** 2011-02-27

**Authors:** Sreela S Namboodiri, Japheth A Opintan, Rebeccah S Lijek, Mercy J Newman, Iruka N Okeke

**Affiliations:** 1 Department of Biology, Haverford College, Haverford, PA 19041, USA; 2 Department of Microbiology, University of Ghana Medical School, Accra, Ghana; 3 University of Maryland Medical School, 655 West Baltimore Street, Baltimore, MD 21201, USA; 4 Department of Microbiology, University of Pennsylvania School of Medicine 3610 Hamilton Walk Philadelphia, PA 19104-6076, USA

## Abstract

**Background:**

Antimicrobial resistance is under-documented and commensal *Escherichia coli *can be used as indicator organisms to study the resistance in the community. We sought to determine the prevalence of resistance to broad-spectrum antimicrobials with particular focus on the quinolones, which have recently been introduced in parts of Africa, including Ghana.

**Results:**

Forty (13.7%) of 293 *E. coli *isolates evaluated were nalidixic acid-resistant. Thirteen (52%) of 2006 and 2007 isolates and 10 (66.7%) of 2008 isolates were also resistant to ciprofloxacin. All but one of the quinolone-resistant isolates were resistant to three or more other antimicrobial classes. Sequencing the quinolone-resistance determining regions of *gyrA *and *parC*, which encode quinolone targets, revealed that 28 quinolone-resistant *E. coli *harboured a substitution at position 83 of the *gyrA *gene product and 20 of these isolates had other *gyrA *and/or *parC *substitutions. Horizontally-acquired quinolone-resistance genes *qnrB1*, *qnrB2*, *qnrS1 *or *qepA *were detected in 12 of the isolates. In spite of considerable overall diversity among *E. coli *from Ghana, as evaluated by multilocus sequence typing, 15 quinolone-resistant *E. coli *belonged to sequence type complex 10. Five of these isolates carried *qnrS1 *alleles.

**Conclusions:**

Quinolone-resistant *E. coli *are commonly present in the faecal flora of Accra residents. The isolates have evolved resistance through multiple mechanisms and belong to very few lineages, suggesting clonal expansion. Containment strategies to limit the spread of quinolone-resistant *E. coli *need to be deployed to conserve quinolone effectiveness and promote alternatives to their use.

## Background

Following emergence of resistance to inexpensive broad-spectrum antimicrobials across much of Africa, quinolone antibacterials have recently been introduced and are widely used. West African studies that sought quinolone resistance in commensal or diarrhoeagenic *Escherichia coli *before 2004 reported no or very low incidences of resistance to nalidixic acid and the fluoroquinolones [1-4]. Thus, available data suggests that resistance to the quinolones was rare in West Africa until the first decade of the 21^st ^century. More recent anecdotal reports and surveillance studies point to emergence of quinolone resistance among enteric pathogens and faecal enteric bacteria in Ghana and elsewhere in West Africa [5-8]. In a study by Nys et al. (2004) faecal isolates of adult volunteers in eight different countries were assessed for susceptibility to antimicrobials in the same laboratory [8]. Resistance to broad spectrum first-generation antibiotics was common and ciprofloxacin resistance was found to be slowly emerging in Asian, South American and African countries, including Ghana [8]. Newman et al. (2004) collected 5099 clinical bacterial isolates (1105 of which were *E. coli*) from nine of the ten regions in Ghana and tested them for antimicrobial susceptibility. They found that over 70% of the isolates were resistant to tetracycline, trimethoprim-sulphamethoxazole, ampicillin and chloramphenicol and reported that 11% of the isolates were ciprofloxacin-resistant [7].

Quinolones inhibit the activity of bacterial DNA gyrase and DNA topoisomerase enzymes, which are essential for replication. Single nucleotide polymorphisms (SNPs) in the quinolone resistance determining regions (QRDR) of *gyrA *and *parC*, the two genes that encode DNA gyrase and topoisomerase IV respectively, can lead to conformational changes in these enzymes that cause them to block quinolones from binding to the DNA- substrate complex, yet still preserve their enzymatic function [9]. In *Escherichia coli *and related Gram-negative bacteria, DNA gyrase is the first target for fluoroquinolones. If *gyrA *has resistance-conferring mutations, the primary target of fluoroquinolone switches from DNA gyrase to topoisomerase IV [10,11]. Studies from other parts of the world have found that resistance-conferring mutations are typically selected in *gyrA *first, and then *parC*.

Although mutations in the QRDR of *gyrA *and *parC *are the most commonly documented resistance mechanisms, resistance has also been known to be conferred by mutations in the second topoisomerase gene, *parE*. Another mechanism of quinolone resistance relies on upregulation of efflux pumps, which export quinolones and other antimicrobials out of the bacterial cell. For example, mutations in the gene encoding a repressor of the *acrAB *pump genes, *acrR*, are associated with quinolone resistance [12]. Quinolone resistance can also be acquired horizontally through transferable quinolone resistance (*qnr*) or other DNA. The Qnr gene product inhibits quinolones binding to target proteins [13]. Other horizontally acquired quinolone resistance genes include *aac(6*'*)-Ib *, encoding a fluroquinolone acetylating enzyme, as well as *qepA *and *oqxAB*, which encode horizontally transmitted efflux pumps [14-16].

Resistance to the quinolones often emerges at low-levels by acquisition of an initial resistance-conferring mutation or gene. Acquisition of subsequent mutations leads to higher levels of resistance to the first-generation quinolone, nalidixic acid and a broadening of the resistance spectrum to include second-generation quinolones (first-generation fluoroquinolones) such as ciprofloxacin, followed by newer second- and third-generation fluoroquinolones [17].

Although multiple mechanisms of quinolone resistance have been reported from other continents, there are few data from sub-Saharan Africa on the molecular basis for quinolone resistance. We performed antimicrobial susceptibility testing on fecal *E. coli *isolates from Accra, Ghana in 2006, 2007 and 2008. We identified isolates that were resistant to nalidixic acid and screened these strains for mutations in the QRDR of *gyrA *and *parC *as well as horizontally-acquired quinolone-resistance genes. In order to gain some insight into resistance dissemination, we also studied inter-strain relatedness among quinolone-resistant *E. coli *isolates by multilocus sequence typing.

## Results

### Resistance to commonly used antimicrobials is high and resistance to the quinolones was detected

**Figure 1:**
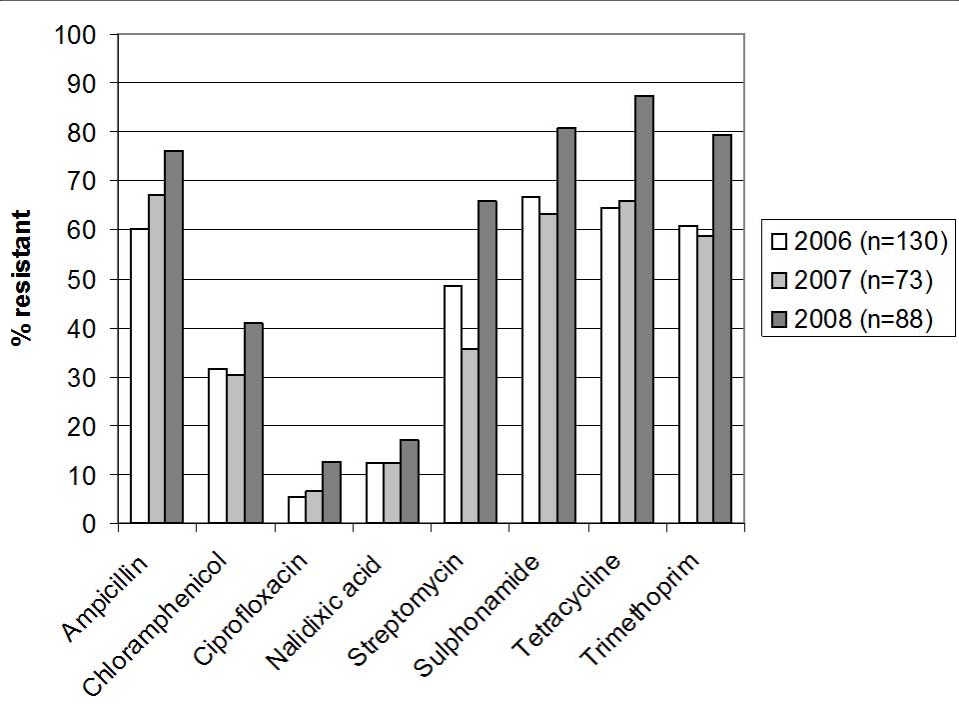
**Proportion of *E. coli *isolates resistant to each of eight broad-spectrum antibacterials in 2006, 2007 and 2008**.

In 2006, 2007 and 2008 respectively, 156, 78 and 101 stool specimens were collected. A total of 293 *Escherichia coli *isolates were recovered from culture of the 335 stool specimens. Consistent with the results of recent studies from West African countries, including Ghana [1,7,8], 50-90% of the *E. coli *isolates were resistant to the broad-spectrum antimicrobials ampicillin, streptomycin, sulphonamides, tetracycline and trimethoprim (Figure 1). Resistance to chloramphenicol was less common but was seen in 30-41% of the isolates. The proportions of isolates resistant to most agents were comparable between 2006 and 2007. However, the proportion of isolates resistant to each antimicrobial in 2008 was significantly greater than those seen in 2006, for all agents (p < 0.05) (Figure 1).

As illustrated in Figure 1 in 2006 and 2007, we recorded resistance rates to nalidixic acid of 12.3% but by 2008, 15 (18.2%) of isolates were nalidixic acid resistant. Ciprofloxacin-resistant isolates represented 7 (5.4%) and 6 (7.7%) of the total number of isolates in 2006 and 2007 respectively. In 2008, 10 (9.9%) of the isolates were fluoroquinolone resistant. Thus, in 2006 and 2007, 13 (52%) quinolone-resistant *E. coli *isolates were ciprofloxacin resistant but in 2008, 10 (67%) of the quinolone-resistant *E. coli *were resistant to ciprofloxacin (Figure 1). Although the numbers were too small to attain statistical significance, organisms with higher nalidixic acid MICs were recovered more commonly in 2008 than in 2006 (Table 1).

**Table 1:** Antimicrobial susceptibility, QRDR mutations and sequence types of quinolone-resistant *E. coli *isolates

Strains	Nali-dixic acid MIC mg/L	Resistance pattern*	QRDR mutations		Horizontally acquired quinolone resistance genes	Allelic profile							Sequence type (ST)	ST complex
							
			Δ GyrA	Δ ParC		*adk*	*fumC*	*gyrB*	*icd*	*mdh*	*purA*	*recA*		
**2006**														

06/073	64	AC NSLTR	none	none	none	6	19	15	16	9	8	7	443	205

06/079	128	AC NSLTR	none	none	none	6	65	2	25	5	5	2	1466	none

06/137	128	AC NSLTR	none	none	none	6	65	2	25	5	5	2	1466	none

06/065	128	A NSLTR	none	none	*qnrS1*	6	7	5	1	8	18	2	206	206

06/036	256	AC NSLTR	none	none	none	6	8	4	1	9	48	7	210	none

06/027	64	C N	S83L	none	*qnrB2*	10	11	4	8	8	8	2	10	10

06/045	64	A NSLTR	S83L	none	none	10	174	4	8	8	8	2	1286	10

06/056	128	A NSLTR	S83L	none	none	112	11	5	12	8	8	86	542	none

06/068	128	AC NSLTR	S83L	none	none	76	43	19	37	30	1	2	1465	none

06/151	>1024	ACPNSLTR	S83L, D87N	none	none	43	41	15	18	11	7	6	101	101

06/154	>1024	ACPN TR	S83L, D87N	none	*qnrB1*	43	41	15	18	11	7	6	101	101

06/030	>1024	ACPN LTR	S83L, D87N	S80I	*qnrS1*	10	11	4	8	8	13	73	617	10

06/110	>1024	ACPNSLTR	S83L, D87N	S80I	none	92	4	87	96	70	58	2	648	none

06/148	>1024	A PNSLTR	S83L, D87N	S80I	none	92	4	87	96	70	58	2	648	none

06/026	>1024	ACPNSLTR	S83L, D87N	S80I, N105S	none	56	6	22	16	11	1	7	455	None

06/112	>1024	ACPN LTR	S83L, D87N	S80I, E84K	none	6	29	32	16	11	8	44	156	156

**2007**					none									

07/16a	256	A PNSLT	none	none	none	6	95	4	18	11	7	14	1304	none

07/34	128	AC N LTR	none	none	none	10	11	4	12	7	8	2	1467	none

07/12	64	NSLT	S83L	none	none	10	11	4	8	8	8	2	10	10

07/37	128	A N L R	S83L	T66I	none	10	11	4	8	8	8	2	10	10

07/SHA	>1024	A PNSLTR	S83L, D87N	none	none	6	11	95	104	8	7	2	450	none

07/375	>1024	A PNSLTR	S83L, D87N	S80I	*qepA*	10	11	4	8	8	8	2	10	10

07/337	>1024	A PN LTR	S83L, D87N	S80I	none	10	11	4	8	8	8	2	10	10

07/282	>1024	ACPNSLTR	S83L, D87N	E84K	*qepA*	6	29	32	16	11	8	44	156	156

07/24A	>1024	ACPNSLTR	S83L, D87N	S80I, E84G	*qnrB1*	85	88	78	29	59	58	62	354	354

**2008**														

08/54	64	AC NSLTR	none	none	none	6	99	22	1	8	7	2	1479	206

08/23	64	ACPNSLTR	none	none	none	10	29	4	8	8	8	44	1473	156

08/43	128	A NSLTR	none	none	none	6	7	14	1	8	18	2	1471	l206

08/33	>1024	ACPNSLTR	none	none	*qnrS1*	6	11	14	8	8	6	6	1469	None

08/91	>1024	ACPNSLTR	none	none	none	10	11	4	1	8	9	2	227	10

08/101	64	A NSLTR	S83A	none	*qnrS1*	10	11	4	1	8	9	2	227	10

08/78	128	ACPNSLTR	S83L	none	*qnrS1*	10	11	4	8	8	8	2	10	10

08/90	256	ACPNSLTR	S83L, D87N	S80I	none	6	4	12	1	20	18	7	410	23

08/11	>1024	A PNSLTR	S83L, D87N	S80I	none	6	6	5	26	11	8	6	1468	none

08/26	>1024	A PN LTR	S83L, D87N	S80I	*qnrS1*	10	11	4	8	8	8	2	10	10

08/87	>1024	A NSLTR	S83L, D87N	S80I	none	10	11	4	8	8	8	2	10	10

08/84	>1024	A NSLTR	S83L, D87N	S80I	*qnrS1*	10	11	4	8	8	8	2	10	10

08/93	>1024	A PNSLTR	S83L, D87N	S80I	none	10	11	4	8	8	8	2	10	10

08/17	>1024	ACPNSLTR	S83L, D87N	S80I, E84V	none	53	40	47	13	36	28	29	131	none

08/39	>1024	ACPNSLTR	S83L, D87N	S80I, A108V	none	92	40	87	96	70	8	29	1470	none

*A = Ampicillin, C = Chloramphenicol, P = Ciprofloxacin, N = Nalidixic acid, S = Streptomycin, L = Sulphonamide, T = Tetracycline R = Trimethoprim

Quinolone resistance was almost always seen in multiply-resistant *E. coli*. As shown in Table 1, all quinolone-resistant *E. coli *(QREC) were resistant to at least one other antimicrobial and all but three of the QREC isolates were resistant to four or more non-quinolone antibacterials. Most QREC demonstrated high-level resistance to nalidixic acid with 21 of 40 of the QREC isolates showing a nalidixic acid MIC that exceeded 1024 mg/L. Among 2006 isolates, low-level resistance was more common, with the MIC_50 _in that year being 128 mg/L. In both 2007 and 2008, the MIC_50 _was >1024 mg/L.

### Quinolone resistant *E. coli *predominantly harbour mutations in *gyrA, parC *or both

Increasing nalidixic acid MICs, accompanied by resistance to fluoroquinolones is often due to the acquisition of multiple mutations in quinolone targets. We sequenced the quinolone-resistance determining regions (QRDRs) of *gyrA *and *parC *in the 40 QREC isolates. As shown in Table 1, 28 (70%) of the quinolone-resistant isolates had at least one non-synonymous substitution in the QRDR of *gyrA *and 18 of these isolates also had one or more non-synonymous mutations in *parC*. Twenty-seven of the 28 isolates with at least one mutation in *gyrA *had a serine to leucine substitution at position 83, one of the most commonly documented resistance conferring mutations [10]. Twenty of these isolates also harboured the frequently documented aspartic acid to asparagine substitution at position 87 and all of these isolates had a nalidixic acid MIC of at least 256 mg/L. Eighteen of them were resistant to ciprofloxacin as well as nalidixic acid.

Eighteen QREC isolates had non-synonymous mutations in the QRDR of *parC *with a serine to isoleucine substitution at position 80, present in 16 strains, being the most common substitution (Table 1). The 2007 isolate with a Thr66Ile substitution in ParC had a single GyrA substitution, Ser83Leu. All other isolates with ParC substitutions also had Ser83Leu and Asp87Asn substitutions in GyrA. Five isolates had more than one ParC substitution. Thr66Ile and Asn105Ser substitutions in ParC, seen in two isolates in this study, have not previously been described in *E. coli *but Thr66Ile has been seen in *Salmonella enterica *serovars Heidelberg and Mbandaka [18](Table 1). Both substitutions occur in strains with other previously described non-synonymous polymorphisms in *parC *and *gyrA*. In each case, the level and spectrum of resistance seen is not significantly greater than that for isolates that lack the novel substitution. Therefore, it is not possible to conclude that either substitution confers additional resistance although confirmation of this assumption, or otherwise, can only be made in isogenic strains.

### Multiple horizontally-transmitted quinlone resistance genes were detected among *E. coli *from Accra

We used PCR to screen for *qnrA, qnrB, qnrS *and *qepA *genes and confirmed all amplicons by sequencing. Of the 40 strains evaluated twelve carried one horizontally acquired quinolone resistance gene. These were *qnrB1 *(2 isolates), *qnrB2 *(1 isolate), *qnrS1 *(7 isolates) and *qepA *(2 isolates). In two isolates, without mutations in *gyrA *and *parC *QRDRs, horizontally-acquired resistance genes could account for the resistance seen. However, in the vast majority of cases, horizontally acquired resistance was seen in combination with QRDR mutations.

### Quinolone-resistant *E. coli *from Accra are over-represented among multi-locus sequence type 10

**Figure 2:**
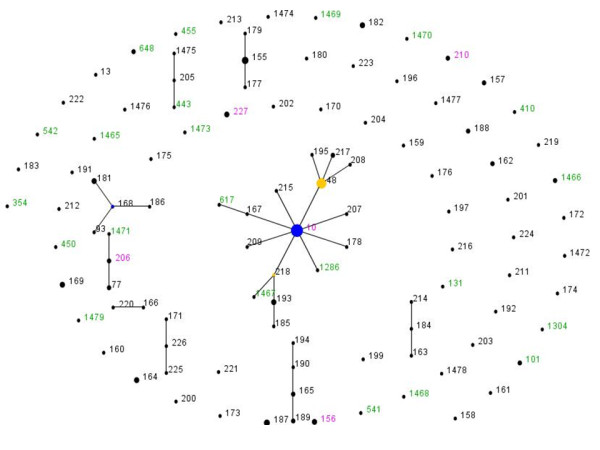
**eBURST output for 165 *E. coli *isolates in the **http://www.mlst.net** database that were isolated in Ghana, including 48 isolates sequence-typed in this study**. Each ST is marked as a dot or node. The size of the node is proportional to the number of isolates contained in that ST. Blue nodes represent predicted founder STs and sub-founders are indicated in yellow. All other STs marked as black dots. STs annotated in green are comprised of quinolone-resistant strains only and those written in pink contain quinolone-sensitive and quinolone-resistant isolates.

We hypothesized that clonal expansion might account, at least in part, for the rise in resistance seen in the course of the study. To test this hypothesis, we subjected all the 40 QREC isolates to multi-locus sequence typing by the scheme of Wirth et al [19] and deposited their allelic profiles in the database at http://www.mlst.net. We identified 30 Sequence Types (STs) among 40 QREC isolates from Ghana (0.75 STs per strain). As shown in Figure 2, quinolone resistance is seen in diverse lineages that have been detected in Ghana. STs that were recovered more than once among the QREC included ST10 (9 isolates) as well as STs101, 156, 227, 648 and 1466 (2 isolates each) (Table 1). Although there were 10 QREC STs that were identified for the first time in this study (reflecting the low proportion of strains from West Africa in the database), only one of these (1466) was seen more than once among QREC (Figure 2, Table 1). Three others were related to STs that were also seen among QREC - ST1471 was a single-locus variant of ST206, and STs1286 and 1467 were respectively single- and double-locus variants of ST10. Horizontally-transmitted quinlone resistance determinants were expectedly detected in strains belonging to multiple STs. However *qnrS1 *alleles were in all but two cases detected among strains belonging to the ST10 complex.

Nine of the 40 QREC isolates obtained in this study belonged to ST10, in contrast to 10 of 125 other *E. coli *from Ghana in the database (p = 0.02, Fisher's exact test). Moreover six other QREC isolates were single- or double- locus variants of ST10. Thus ST10 appeared to be over-represented among QREC isolates from Ghana. Because most of the isolates from Ghana were deposited in the database two to four years in advance of our own study, we sequence typed eight non-QREC isolates selected at random from our 2008 isolates. All eight belonged to different sequence types (10, 349, 541, 1474, 1475, 1476, 1477 and 1478), five of the eight sequence types were novel, and only one 2008 non-QREC strain was an ST10 isolate. Therefore our data suggest that ST10-complex QREC may represent a successful quinolone-resistant lineage.

## Discussion

Evolution of reduced susceptibility to the quinolones is causing concern following rapidly rising rates of fluoroquinolone-resistant *E. coli *in many parts of the world [20]. In African countries with a high infectious disease burden, formal and informal health systems depend heavily on broad spectrum orally-administrable antibacterials. In this study, we found that most commensal *E. coli *isolates are resistant to ampicillin, sulphonamides, tetracycline and trimethoprim, as well as streptomycin, which have been used to treat actual and supposed bacterial infections in Ghana for over four decades, and that resistance to these agents is increasing with time. We also found that about a third of isolates were resistant to chloramphenicol. Fluoroquinolone antimicrobials have been recently introduced as an effective alternative to older antibacterials that have been compromised by resistance. However, although resistance rates were markedly lower for this class of drugs, we also found that quinolone resistance was increasingly common among fecal *E. coli *in this study.

We determined that 12-18% of fecal *E. coli *isolated from healthy individuals in Accra in 2006, 2007 and 2008 are quinolone resistant. Twenty-three of the 40 QREC isolated were resistant to the fluoroquinolone ciprofloxacin. Ciprofloxacin-resistant QREC, showing high-level nalidixic acid resistance, were more commonly isolated in 2008 than in 2006 and 2007. Strains with one or no mutations in *gyrA *were typically ciprofloxacin sensitive. However most isolates had accumulated a second *gyrA *mutation and/or mutations in *parC *and were fluoroquinolone resistant. The QRDR polymorphisms most commonly detected in this study are those most frequently reported in the literature [10]. As has been validated experimentally in isogenic strains, high-level nalidixic acid resistance and fluoroquinolone resistance in isolates in this study was associated with *parC *substitutions in strains also harbouring substitutions in *gyrA *[17]. However, *gyrA *and *parC *mutations did not absolutely correlate with nalidixic acid MICs, partly due to horizontally-acquired quinolone-resistance genes. We sought *qnrA, qnrB, qnrS *and *qepA *genes by PCR and confirmed all amplicons by sequencing. We found that two isolates without mutations in the QRDRs of *gyrA *and *parC*, as well as ten isolates with QRDR mutations carried a *qnrS1*, a *qnrB *or a *qepA *allele. The presence of horizontally-acquired genes accounted in part for elevated nalidixic acid MICs in strains that harboured these genes, but not completely. It is therefore possible that other resistance mechanisms, such as ParE polymorphisms, other horizontally acquired resistance genes (such as *oqxAB *and *aac(6*'*)-Ib *for example), over-active efflux, or even novel mechanisms are present in some of the isolates.

Resistance patterns in pathogens often mirror those in commensals. This is borne out by our recent documentation of quinolone resistance in *Vibrio cholerae *isolates recovered in the same time frame as the *E. coli *strains presented in this report [21]. Fifteen of the 40 QREC isolates identified in this study belonged to ST10, or were single- or double-locus variants of this ST, pointing to the possibility of clonal expansion. ST10-complex strains were isolated in all three years and therefore over-representation of these STs in our sample cannot be explained by short-term, localized clustering. There are four major *E. coli *phylogenetic clades: ECOR A, B1, B2 and D. Few studies have looked at the geographical variance in the distribution of these groups but overall, QREC from Ghana were predominantly drawn from ECOR group A. Of the STs identified in this study that are classified into ECOR clades at the *E. coli *MLST database, ST10 complex (14 isolates) belong to ECOR group A, ST131 (1 isolate) to ECOR B2, STs101 and 410 (3 isolates) to ECOR B1 and STs 156, 206 and 210 (4 isolates) are hybrids of ECOR A and B1, that is AxB1. Available data appear to suggest that ECOR A strains are highly prevalent in Africa, compared to some other world regions [22]. However, when we compared the sequence types of quinolone-resistant and -susceptible strains from Ghana only, we still found that resistant strains were over-represented in the ST10 complex. Pandemic clonal expansion of some QREC lineages has been reported in the literature [23-28]. For example, ST131 is a globally disseminated multi-resistant clone and was detected once among the QREC in this study. Recent reports suggest that isolates from Europe and North America that belong to ST10- or ST131- clonal complexes may be less likely to carry virulence factors for invasive disease, but more likely to be fluoroquinolone resistant [24-28]. However it is equally likely that mutations to fluoroquinolone resistance are more likely to be stably inherited in a specific genetic background. Our own data also appear to suggest that, although horizontally acquired, *qnrS1 *is associated with ST10 complex.

A recent paper by Davidson et al suggests that the antimalarial chloroquine may select for fluoroquinolone-resistant fecal bacteria in malaria endemic areas and proposes that chloroquine-mediated selection accounts for high levels of QREC in fecal flora in villages in South America [29]. However, data from within Africa (where chloroquine has seen heaviest use) to support this hypothesis are currently lacking and evidence from elsewhere supports a link between quinolone resistance rates in *E. coli *and fluoroquinolone consumption at population levels [30,31]. Our own data suggest that even if resistance was present at low levels prior to the introduction of the quinolones, an upsurge in resistance may reflect a selective advantage that came with quinolone introduction. This is particularly likely for Ghana were artemisinin combination therapies have recently been introduced to replace chloroquine. Furthermore, because almost all quinolone resistant-*E. coli *were multiply resistant, selective pressure from other, even more commonly applied, antimicrobials will help to maintain the quinolone-resistant clonal groups we identified in this study.

## Conclusion

Fluoroquinolones, largely ciprofloxacin, were introduced very recently to Ghana with high expectations. This study demonstrates that resistance to these drugs is already common and occurs through multiple mechanisms, suggesting that heavy use of these valuable drugs may rapidly obliterate their usefulness. In addition to the impact that the emergence and dissemination of quinolone resistant bacteria may have on the use of fluoroquinolone antibacterials, we found that QREC were almost invariably resistant to multiple antimicrobials. This is worrisome because it means that, if the commensal flora is reflective of resistance profiles in pathogens, there may be few low-cost alternatives for managing infections due to Gram-negative enteric organisms. Additionally, horizontally-acquired resistance to the quinolones, and presumably other agents may be present on mobile elements that could be transmitted to pathogens. Recent calls for antimicrobial development have spotlighted hospital pathogens and Gram-positive community-acquired pathogens such as Staphylococci [32]. Our data suggest that there is also a pressing need for orally administrable drugs with activity against Gram-negative organisms, which can be used to manage community enteric infections in Ghana and other parts of Africa. Additionally, known strategies for containing antimicrobial resistance need to be more rigorously applied [33-35].

## Methods

### Strains

This study was approved by the Institutional Review Board of the University of Ghana Medical School. *E. coli *isolates were recovered from stool specimens collected from consenting, apparently healthy individuals who presented for medical check-ups at the Korle-Bu Teaching Hospital and the Microbiology Department of the University of Ghana Medical School. Colonies with a typical *E. coli *morphology on MacConkey agar were subjected to biochemical testing, and where this was inconclusive, by 16 S amplification and sequencing [36]. Colonies from the same specimen with identical biochemical and susceptibility profiles were treated as identical strains.

### Antimicrobial susceptibility testing

Each isolate was tested for susceptibility to eight antimicrobials using the Clinical and Laboratory Standards Institute (CLSI, formerly NCCLS) disc diffusion method [37]. Antimicrobial discs and control strain *E. coli *ATCC 35218 were obtained from Remel. The antimicrobial discs used contained ampicillin (10 μg), streptomycin (10 μg), trimethoprim (5 μg), tetracycline (30 μg), nalidixic acid (30 μg), chloramphenicol (30 μg), ciprofloxacin (5 μg) and sulphonamide (300 μg). Inhibition zone diameters were interpreted in accordance with CLSI guidelines with WHONET software version 5.3 [38]. Minimum inhibitory concentrations (MICs) to nalidixic acid were measured using the agar dilution technique on Mueller-Hinton agar as recommended by the CLSI and using *E. coli *ATCC 35218 as control [39].

### Mutational analysis of the Quinolone-Resistance Determining Regions of *gyrA *and *parC*

DNA was extracted from each quinolone-resistant isolate, using the Promega Wizard genomic extraction kit. The QRDR of the *gyrA *and *parC *genes were amplified from DNA templates by PCR using Platinum PCR supermix (Invitrogen) and the primer pairs listed in Table 2. PCR reactions began with a two-minute hot start at 94°C followed by 30 cycles of 94°C for 30 s, annealing temperature, 30 s and 72°C for 30 s. *gyrA *amplifications were annealed at 58°C and *parC *reactions were annealed at 52°C. *E. coli *K-12 MG1655 [40] was used as a control. Amplicons were sequenced on both strands and predicted peptide sequences were compared to the corresponding gene from the MG1655 genome [40] by pair-wise FASTA alignments.

**Table 2:** Oligonucleotide primers used in this study

Target gene	Primer	Primer Sequence	Purpose	Reference
*gyrA*	gyrA12004	TGC CAG ATG TCC GAG AT	*gyrA *QRDR amplification	[12]
			
	gyrA11753	GTA TAA CGC ATT GCC GC		

*parC*	EC-PAR-A	CTG AAT GCC AGC GCC AAA TT	*parC *QRDR amplification	[43]
			
	EC-PAR-B	GCG AAC GAT TTC GGA TCG TC		

*qnrA*	qnrA-1A	TTC AGC AAG ATT TCT CA	*qnrA *detection	[42]
			
	qnrA-1B	GGC AGC ACT ATT ACT CCC AA		

*qnrB*	qnrB-CS-1A	CCT GAG CGG CAC TGA ATT TAT	*qnrB *detection	[42]
			
	qnrB-CS-1B	GTT TGC TGC TCG CCA GTC GA		

*qnrS*	qnrS-1A	CAA TCA TAC ATA TCG GCA CC	*qnrS *detection	[42]
			
	qnr-1B	TCA GGA TAA ACA ACA ATA CCC		

*qepA*	qepA-F	GCAGGTC CAGCAGCGGGTAG	*qepA *detection	[41]
			
	qepA-R	CTTCCTGCCCGAGTATC GTG		

*adk*	*adk*F	ATTCTGCTTGGCGCTCCGGG	MLST	[19]
			
	*adk*R	CCGTCAACTTTCGCGTATTT		

*fumC*	*fumC*F	TCACAGGTCGCCAGCGCTTC	MLST	[19]
			
	*fumC*R	GTACGCAGCGAAAAAGATTC		

*gyrB*	*gyrB*F	TCGGCGACACGGATGACGGC	MLST	[19]
			
	*gyrB*R	ATCAGGCCTTCACGCGCATC		

*icd*	*icd*F	ATGGAAAGTAAAGTAGTTGTTCCGGCACA	MLST	[19]
			
	*icd*R	GGACGCAGCAGGATCTGTT		

*mdh*	*mdh*F	ATGAAAGTCGCAGTCCTCGGCGCTGCTGGCGG	MLST	[19]
			
	*mdh*R	TTAACGAACTCCTGCCCCAGAGCGATATCTTTCTT		

*purA*	*purA*F	CGCGCTGATGAAAGAGATGA	MLST	[19]
			
	*purA*R	CATACGGTAAGCCACGCAGA		

*recA*	*recA*F	CGCATTCGCTTTACCCTGACC	MLST	[19]
			
	*recA*R	TCGTCGAAATCTACGGACCGGA		

MLST - multi-locus sequence typing; QRDR - quinolone-resistance determining region

### Identification of horizontally-acquired quinolone-resistance genes

Horizontally-acquired quinolone-resistance genes were identified by PCR. The primers of Liu et al [41] were used to screen for the *qepA *gene and *qnrA*, *qnrB*, and *qnrS *were identified with PCR using the primer pairs published by Wu et al [42] (Table 2). Amplicons were sequence-verified.

### Multi-locus sequence typing

Gene fragments from the *adk, fumC, gyrB, icd, mdh, purA *and *recA *were amplified using primers listed in Table 2, as described by Wirth et al [19]. Amplified DNA products were sequenced from both ends. Allele assignments were made at the publicly accessible *E. coli *MLST database at http://www.mlst.net. Phylogenetic inferences about ancestral allelic profiles and strain interrelatedness were made using eBURSTv3 at http://eburst.mlst.net/ defining clonal complexes based on groups sharing five identical alleles and bootstrapping with 1000 samplings.

### Statistical analysis

Proportions were compared using the χ^2 ^or Fisher's exact test with p-values less than 0.05 being considered significant.

## Authors' contributions

SSN performed molecular experiments, analysed and interpreted data, and contributed to writing the paper. JAO collected isolates and performed microbiology experiments. RSL designed and performed molecular experiments. MJN co-conceived the study and collected isolates. INO co-conceived the study, performed microbiology and molecular experiments, analysed and interpreted data and wrote the manuscript. All authors read and approved the final manuscript.

## Funding

This work was supported by a Branco Weiss Fellowship from the Society in Science, ETHZ, Zürich to INO. SSN and RSL were HHMI-supported undergraduate researchers, and RSL was also an Arnold and Mabel Beckman Scholar, at Haverford College.

## References

[B1] Okeke IN Fayinka ST Lamikanra A Antibiotic resistance trends in *Escherichia coli *from apparently healthy Nigerian students (1986-1998) Emerg Infect Dis 2000 6 4 393 396 10.3201/eid0604.000413 10905975 PMC2640895

[B2] Mendez Arancibia E Pitart C Ruiz J Marco F Gascon J Vila J Evolution of antimicrobial resistance in enteroaggregative *Escherichia coli *and enterotoxigenic *Escherichia coli *causing traveller's diarrhoea J Antimicrob Chemother 2009 64 2 343 347 10.1093/jac/dkp178 19474067

[B3] Okeke IN Lamikanra A Czeczulin J Dubovsky F Kaper JB Nataro JP Heterogeneous virulence of enteroaggregative *Escherchia coli *strains isolated from children in Southwest Nigeria J Infect Dis 2000 181 252 260 10.1086/315204 10608774

[B4] Okeke IN Steinruck H Kanack KJ Elliott SJ Sundstrom L Kaper JB Lamikanra A Antibiotic-resistant cell-detaching *Escherichia coli *strains from Nigerian children J Clin Microbiol 2002 40 1 301 305 10.1128/JCM.40.1.301-305.2002 11773139 PMC120082

[B5] Soge OO Adeniyi BA Roberts MC New antibiotic resistance genes associated with CTX-M plasmids from uropathogenic Nigerian *Klebsiella pneumoniae* J Antimicrob Chemother 2006 58 5 1048 1053 10.1093/jac/dkl370 16997844

[B6] Nabeth P Perrier-Gros-Claude J-D Juergens-Behr A Dromigny J-A In vitro susceptibility of quinolone-resistant Enterobacteriaceae uropathogens to fosfomycin trometamol, in Dakar, Senegal Scand J Infect Dis 2005 37 6 497 499 10.1080/00365540510038956 16012011

[B7] Newman MJ Frimpong E Asamoah-Adu A Sampane-Donkor E Opintan JA Resistance to antimicrobial drugs in Ghana The Ghanaian-Dutch Collaboration for Health Research and Development 2004

[B8] Nys S Okeke IN Kariuki S Dinant GJ Driessen C Stobberingh EE Antibiotic resistance of faecal *Escherichia coli *from healthy volunteers from eight developing countries J Antimicrob Chemother 2004 54 5 952 955 10.1093/jac/dkh448 15471998

[B9] Hawkey PM Mechanisms of quinolone action and microbial response J Antimicrob Chemother 2003 51 Suppl 1 29 35 10.1093/jac/dkg207 12702701

[B10] Hopkins KL Davies RH Threlfall EJ Mechanisms of quinolone resistance in *Escherichia coli *and *Salmonella*: recent developments Int J Antimicrob Agents 2005 25 5 358 373 10.1016/j.ijantimicag.2005.02.006 15848289

[B11] Hooper DC Mechanisms of action of antimicrobials: focus on fluoroquinolones Clin Infect Dis 2001 32 Suppl 1 S9 S15 10.1086/319370 11249823

[B12] Wang H Dzink-Fox JL Chen M Levy SB Genetic characterization of highly fluoroquinolone-resistant clinical Escherichia coli strains from China: role of *acrR *mutations Antimicrob Agents Chemother 2001 45 5 1515 1521 10.1128/AAC.45.5.1515-1521.2001 11302820 PMC90498

[B13] Tran JH Jacoby GA Mechanism of plasmid-mediated quinolone resistance Proc Natl Acad Sci USA 2002 99 8 5638 5642 10.1073/pnas.082092899 11943863 PMC122823

[B14] Hansen LH Jensen LB Sørensen HI Sørensen SJ Substrate specificity of the OqxAB multidrug resistance pump in *Escherichia coli *and selected enteric bacteria J Antimicrob Chemother 2007 60 1 145 147 10.1093/jac/dkm167 17526501

[B15] Yamane K Wachino J-i Suzuki S Kimura K Shibata N Kato H Shibayama K Konda T Arakawa Y New plasmid-mediated fluoroquinolone efflux pump, QepA, found in an *Escherichia coli *clinical isolate Antimicrob Agents Chemother 2007 51 9 3354 3360 10.1128/AAC.00339-07 17548499 PMC2043241

[B16] Strahilevitz J Jacoby GA Hooper DC Robicsek A Plasmid-mediated quinolone resistance: a multifaceted threat Clin Microbiol Rev 2009 22 4 664 689 10.1128/CMR.00016-09 19822894 PMC2772364

[B17] Morgan-Linnell SK Zechiedrich L Contributions of the combined effects of topoisomerase mutations toward fluoroquinolone resistance in *Escherichia coli* Antimicrob Agents Chemother 2007 51 11 4205 4208 10.1128/AAC.00647-07 17682104 PMC2151436

[B18] Eaves DJ Randall L Gray DT Buckley A Woodward MJ White AP Piddock LJV Prevalence of mutations within the quinolone resistance-determining region of *gyrA, gyrB, parC*, and *parE *and association with antibiotic resistance in quinolone-resistant *Salmonella enterica* Antimicrob Agents Chemother 2004 48 10 4012 4015 10.1128/AAC.48.10.4012-4015.2004 15388468 PMC521866

[B19] Wirth T Falush D Lan R Colles F Mensa P Wieler LH Karch H Reeves PR Maiden MC Ochman H Sex and virulence in *Escherichia coli: *an evolutionary perspective Mol Microbiol 2006 60 5 1136 1151 10.1111/j.1365-2958.2006.05172.x 16689791 PMC1557465

[B20] Livermore DM Has the era of untreatable infections arrived? J Antimicrob Chemother 2009 64 Suppl 1 i29 36 10.1093/jac/dkp255 19675016

[B21] Opintan JA Newman MJ Nsiah-Poodoh OA Okeke IN *Vibrio cholerae *O1 from Accra, Ghana carrying a class 2 integron and the SXT element J Antimicrob Chemother 2008 62 5 929 933 10.1093/jac/dkn334 18755696 PMC2566517

[B22] Robinson DA Falush D Feil EJ Bacterial population genetics in infectious disease 2010 Hoboken, N.J.: J. Wiley

[B23] Uchida Y Mochimaru T Morokuma Y Kiyosuke M Fujise M Eto F Eriguchi Y Nagasaki Y Shimono N Kang D Clonal spread in Eastern Asia of ciprofloxacin-resistant *Escherichia coli *serogroup O25 strains, and associated virulence factors Int J Antimicrob Ag 2010 35 5 444 450 10.1016/j.ijantimicag.2009.12.012 20188525

[B24] Johnson James R Kuskowski Michael A Owens K Gajewski A Winokur Patricia L Phylogenetic origin and virulence genotype in relation to resistance to fluoroquinolones and/or extended spectrum cephalosporins and cephamycins among *Escherichia coli *isolates from animals and humans J Infect Dis 2003 188 5 759 768 10.1086/377455 12934193

[B25] Moreno E Prats G Sabate M Perez T Johnson JR Andreu A Quinolone, fluoroquinolone and trimethoprim/sulfamethoxazole resistance in relation to virulence determinants and phylogenetic background among uropathogenic *Escherichia coli* J Antimicrob Chemother 2006 57 2 204 211 10.1093/jac/dki468 16390858

[B26] Johnson JR Menard M Johnston B Kuskowski MA Nichol K Zhanel GG Epidemic clonal groups of *Escherichia coli *as a cause of antimicrobial-resistant urinary tract infections in Canada, 2002 to 2004 Antimicrob Agents Chemother 2009 53 7 2733 2739 10.1128/AAC.00297-09 19398649 PMC2704706

[B27] Grude N Strand L Mykland H Nowrouzian FL Nyhus J Jenkins A Kristiansen BE Fluoroquinolone-resistant uropathogenic *Escherichia coli *in Norway: evidence of clonal spread Clin Microbiol Infect 2008 14 5 498 500 10.1111/j.1469-0691.2008.01952.x 18294242

[B28] Boyd L Atmar R Randall G Hamill R Steffen D Zechiedrich L Increased fluoroquinolone resistance with time in *Escherichia coli *from >17,000 patients at a large county hospital as a function of culture site, age, sex, and location BMC Infectious Diseases 2008 8 1 4 10.1186/1471-2334-8-4 18197977 PMC2258293

[B29] Davidson RJ Davis I Willey BM Rizg K Bolotin S Porter V Polsky J Daneman N McGeer A Yang P Antimalarial therapy selection for quinolone resistance among *Escherichia coli *in the absence of quinolone exposure, in tropical South America PLoS ONE 2008 3 7 e2727 10.1371/journal.pone.0002727 18648533 PMC2481278

[B30] van de Sande-Bruinsma N Grundmann H Verloo D Tiemersma E Monen J Goossens H Ferech M Antimicrobial drug use and resistance in Europe Emerg Infect Dis 2008 14 11 1722 1730 10.3201/eid1411.070467 18976555 PMC2630720

[B31] Gottesman BatÂ S Carmeli Y Shitrit P Chowers M Impact of quinolone restriction on resistance patterns of *Escherichia coli *isolated from urine by culture in a community setting Clin Infect Dis 2009 49 6 869 875 19686074 10.1086/605530

[B32] Talbot GH Bradley J Edwards JE Gilbert D Scheld M Bartlett JG Bad bugs need drugs: An update on the development pipeline from the antimicrobial availability task force of the Infectious Diseases Society of America Clin Infect Dis 2006 42 5 657 668 10.1086/499819 16447111

[B33] Okeke IN Klugman KP Bhutta ZA Duse AG Jenkins P O'Brien TF Pablos-Mendez A Laxminarayan R Antimicrobial resistance in developing countries. Part II: strategies for containment Lancet Infect Dis 2005 5 9 568 580 10.1016/S1473-3099(05)70217-6 16122680

[B34] Okeke IN Aboderin AO Byarugaba DK Ojo O Opintan JA Growing problem of multidrug-resistant enteric pathogens in Africa Emerg Infect Dis 2007 13 11 1640 1646 18217545 10.3201/eid1311.070674 PMC3375797

[B35] Nugent R Okeke IN When medicines fail: recommendations for curbing antibiotic resistance J Infect Dev Ctries 2010 4 6 355 356 20601785

[B36] Lane DJ Stackebrandt E, Goodfellow M 16S/23 S rRNA sequencing Nucleic Acid Techniques in Bacterial Systematics 1991 New York: John Wiley and Sons 115 175

[B37] NCCLS Performance standards for antimicrobial disk susceptibility tests, 8th Edition; Approved standard 2003 Villanova, PA: National Committee for Clinical Laboratory Standards 130

[B38] O'Brien TF Stelling JM WHONET: an information system for monitoring antimicrobial resistance Emerg Infect Dis 1995 1 2 66 10.3201/eid0102.950209 PMC2626837 8903165

[B39] CLSI Methods for dilution antimicrobial susceptiblity tests for bacteria that grow aerobically, 7th Edition; Approved standard 2006 Wayne, PA: Clinical and Laboratory Standards Institute

[B40] Blattner FR Plunkett G Bloch CA Perna NT Burland V Riley M Collado-Vides J Glasner JD Rode CK Mayhew GF The complete genome sequence of *Escherichia coli *K-12 Science 1997 277 5331 1453 1474 10.1126/science.277.5331.1453 9278503

[B41] Liu J-H Deng Y-T Zeng Z-L Gao J-H Chen L Arakawa Y Chen Z-L Coprevalence of plasmid-mediated quinolone resistance determinants QepA, Qnr, and AAC(6')-Ib-cr among 16 S rRNA methylase RmtB-producing *Escherichia coli *isolates from pigs Antimicrob Agents Chemother 2008 52 8 2992 2993 10.1128/AAC.01686-07 18490500 PMC2493129

[B42] Wu J-J Ko W-C Tsai S-H Yan J-J Prevalence of plasmid-mediated quinolone resistance determinants QnrA, QnrB, and QnrS among clinical isolates of *Enterobacter cloacae *in a Taiwanese hospital Antimicrob Agents Chemother 2007 51 4 1223 1227 10.1128/AAC.01195-06 17242140 PMC1855486

[B43] Deguchi T Yasuda M Nakano M Ozeki S Kanematsu E Nishino Y Ishihara S Kawada Y Detection of mutations in the *gyrA *and *parC *genes in quinolone-resistant clinical isolates of *Enterobacter cloacae* J Antimicrob Chemother 1997 40 4 543 549 10.1093/jac/40.4.543 9372424

